# Urban–rural difference in the costs of disability and its effects on poverty among people with disabilities in China

**DOI:** 10.3389/fpubh.2022.989540

**Published:** 2022-11-25

**Authors:** Juan Liao, Qi Wang, Jin-Ling Huang, Ya-Min Wei

**Affiliations:** ^1^School of Management, Capital Normal University, Beijing, China; ^2^School of Management, Beijing Union University, Beijing, China; ^3^School of Management, Hebei University, Baoding, China; ^4^Department of Gender Studies, London School of Economics and Political Science, London, United Kingdom

**Keywords:** costs of disability, standard of living, urban-rural gap, poverty, China

## Abstract

The urban–rural difference in poverty is an important issue in China, particularly for people with disabilities. The extra costs of disability render this population susceptible to falling into poverty, where this can exacerbate the inequality among people with disabilities between urban and rural areas of the country. Previous studies have provided empirical evidence for the extra costs of disabilities in certain countries, but little scholarly attention has been devoted to the urban–rural gap in the costs of disability, particularly in countries like China that have a dual urban–rural system. This study explores changes in the extra costs of disability in China between urban and rural households with disabled members from 2008 to 2018 by using the standard of living approach. We apply the Foster–Greer–Thorbecke Poverty Index to measure the rates of poverty in urban and rural households with disabilities after considering the costs of disability. The results reveal that the costs of disability were not always lower for rural households than for urban households. At the same time, many rural households with disabled people were found to suffer from severe poverty owing to the high costs of their disabilities. The difference in health insurance and rehabilitation services between urban and rural China have led to an urban-rural gap in the costs of disability. This suggests that supplying more goods and services for disabled people in rural areas, especially free services, and raising the reimbursement due to them from their health insurance can help improve their standard of living.

## Introduction

The difference in the levels of urban and rural development is an important cause of inequality in a country, and the resulting gap in poverty between urban and rural areas is a significant issue in China as well. The urban–rural gap in poverty among disabled persons is notable, and there are major inequalities in their health status because they have varying degrees of access to healthcare (1). People with disabilities experience unequal access to healthcare in urban and rural areas, and thus incur different costs that are likely to exacerbate income inequality and impoverish disabled people in rural areas. In general, a large portion of the extra costs of disability is accounted for by healthcare services. However, there are clear urban–rural differences in these services and their costs in China. First, the availability of medical resources in China's urban and rural areas is significantly different, and high-quality medical resources are concentrated in cities. Many disabled people in rural areas thus do not have access to appropriate healthcare services. If these people have few opportunities to access healthcare services, they may spend less on them such that the extra costs of disability may appear to be lower those for urban disabled people. Demand is stifled by the poor healthcare services in rural areas. Second, there is a “reimbursement divide” in health insurance between urban and rural residents in China. The reimbursement rate for healthcare expenses is higher for urban residents than for rural residents. If disabled people in urban and rural areas receive the same healthcare services, the latter usually pay more for them than the former due to the particularities of the health insurance schemes. Therefore, for a country like China that has a dual urban–rural system, it is particularly important to study the extra costs of disability and their impact on the poverty of disabled people in urban and rural areas.

Eradicating poverty is an important target of the 2030 Agenda on Sustainable Development. People with disabilities (PWD) worldwide and their families are more likely to fall into poverty than the average person (2). Regardless of how poverty is defined, it is always tied to disability (3). Disabled persons accounted for 18% of people suffering from poverty in China in 2019 (4). The Chinese government has made concerted efforts in recent years to reduce poverty, especially in rural areas. The government has been developing an administrative database since 2013 to better identify the segment of the population most in need of targeted interventions and the type of assistance that can best alleviate its situation. Officials can use this information to construct targeted measures to reduce poverty. A person is qualified to receive benefits if their per capita family income is below the national poverty line. However, a problem with this approach to income to measure poverty among PWD is that it fails to account for the extra costs of their disabilities. The latest poverty line was set by the National Bureau of Statistics of China in 2011. It refers to rural residents with per capita income below CNY 2,300 per year (unchanged prices from 2010) because a majority of China's poor people live in rural areas. According to this standard for the poverty line, a total 4.13 million poor and disabled people in rural areas were registered by the Poverty Alleviation Office of the State Council in 2015. However, data from the National Special Survey on the Status and Needs of Basic Services for Disabled People conducted by the China Disabled Persons' Federation in 2015, showed that nearly 10 million rural disabled people still live in poverty (5). This means that this measure of poverty had excluded approximately 6 million poor disabled persons in rural China from the government's records in 2015. Therefore, a significant number of poor disabled persons are not being covered by the government's poverty reduction policies and thus cannot benefit from its poverty alleviation programs. Precisely identifying and measuring the status of poverty among disabled people is vital as it can help determine the underlying reasons for their situation so that the necessary corrective measures can be taken.

Measuring poverty among disabled persons is closely related to the extra costs of disability. However, China currently measures poverty based on per capita household income, without considering the costs of disability. On the premise of guaranteeing compulsory education, basic health care, and housing, China's poverty line makes two assumptions: First, spending on food can not only help meet people's basic needs (2,100 calories per person per day) of survival, but can also satisfy their daily protein requirement of about 60 grams per person per day. Second, spending on food accounts for 60% of the income of people on the poverty line to guarantee a certain amount of spending on items other than food. The current poverty line in China's rural areas represents a standard of living with adequate provision of food and clothing (6). Households with PWD have a lower standard of living than those without PWD given that they have the same household income. Therefore, some households with PWD that are actually below the poverty line are not included in the poverty-related statistics. Because poverty is measured without considering the extra costs of disability, the number of poor and disabled people in China is actually higher than is reflected by government records. According to the Second National Sample Survey of Disabled Persons, the total number of PWD in China is 82.96 million. Three quarters of them, more than 60 million, live in rural areas, where most of China's poor are concentrated. We expect that many poor and disabled people are still not identified in the statistics and measures used by the government.

Previous research on the extra costs of disability has largely focused on high-income countries, and less attention has been paid to the situation in low- and middle-income countries. The urban–rural difference is often large in developing countries, but has not attracted sufficient attention in prevalent works. For instance, Antón et al. (7), and Morris and Zaidi (8) compared the extra costs of disabilities in European countries. Many studies have been conducted on the United Kingdom (9–11), Ireland (12, 13), Australia (14–16) and United States (17, 18). A few articles have addressed this subject in low- and middle-income countries, including Vietnam (19), Cambodia (20), Turkey (21), and Ghana (22), but few studies have considered the extra costs of disability in China. Wang et al. (23) focused on the extra costs incurred by older people with disabilities in urban areas in northern China. Loyalka et al. (24) showed that the costs of disability were higher for urban households with disabled members than for rural households, but did not relate this estimation to the rates of poverty among urban and rural households with disabled members. In addition, the estimation of costs in the previous two papers cited was based on outdated datasets and cross-sectional data from 2006 that cannot reflect the changes in these costs over time or the more recent situation in China. Little research has been devoted to urban–rural differences in the extra costs of disability. One such study on China showed that the additional costs incurred by urban households with PWD were higher than those incurred by rural households with PWD in 2006 (7). A study on Cambodian that used mixed cross-sectional data from 2009 to 2014 yielded similar results (11). Both studies were cross-sectional, however, and did not reflect trends over time.

Many researchers have separately explored the costs of disability and poverty (12, 13, 15, 16, 21, 25), with few choosing to evaluate the two topics together. Even if some studies combine the extra costs of disability with poverty, few considered the impact of the rural–urban gap in the costs of disability on poverty. For example, Saunders (14) explored the costs of disability and the incidence of poverty in Australia, and Zaidi and Burchardt (9) found that the rate of poverty of a household increased after accounting for the costs of disability in the U.K. Morris and Zaidi (8) compared the rates of poverty among disabled people before and after adjusting for the costs of disability in 15 European countries, and found that the mean increased from 43 to 68%. Palmer et al. (20) showed the extra costs of disability were lower than those in urban areas, and that households with at least one disabled member in urban Cambodia had higher poverty rates than those in rural areas when the extra costs of disability were taken into account. However, whether this situation obtains in developing countries with a dual urban–rural system has not been investigated in the relevant research to date. For instance, research on China has estimated only the rural–urban difference in the extra costs of disability, but has not linked it to poverty among disabled people in urban and rural areas.

Few studies have examined the temporal trends of the extra costs of disability. Most previous studies use cross-sectional data from a single year or mixed cross-sectional data. A few studies used panel data (12, 15, 16), but have mainly involved estimations based on econometric models without examining the temporal trend of extra costs of disability. This trend in developing countries may reflect problems with relevant policies, and provide an empirical basis for improve them.

China's economy has developed rapidly in the past few decades and people's wellbeing has accordingly risen, but significant differences in prosperity persist between urban and rural areas. On the one hand, although the income of disabled people has increased year by year, the gap in income between people with and without disabilities is widening, and the income of urban households with PWD is much higher than that of their rural counterparts (26). On the other hand, the income gap between urban and rural PWD is also increasing (27). Research on income-related inequality in the context of health-care utilization has shown that the use of medical services in China is unequal. The richer the people are, the greater is the number of healthcare services that they use (28). In the context of health insurance, the New Cooperative Medical Scheme (NCMS) covering China's rural population has failed to alleviate the urban–rural gap in medical treatment, and rural residents still face a heavy economic burden when they fall ill (29). The poorer a family is, the heavier is the burden of medical expense on it, and is heavier for poor rural families than that urban families (30). Although many studies have examined the disparity in medical costs between urban and rural residents, the urban–rural gap between households with PWD and the extra costs of disability have not been extensively investigated in this context. Our article advances this research to examine changes in the costs of disability over time, and relates these costs to the rates of poverty among urban and rural households with PWD in China.

In summary, the past research focused on developed countries, and has rarely attended to the urban–rural gap in the extra costs of disability and its relationship with health insurance. An analysis of the change in these extra costs over time is also lacking. This study aims to address this gap in the literature. We apply the standard of living (SoL) approach by using three time points in survey data to study changes in the urban–rural divide in the extra costs of disabilities over time, and examines the rates of poverty among urban and rural households in China containing members with disabilities. We also explore the relation between the costs of disability and health insurance, and seek ways to narrow the urban–rural gap in them in developing countries like China that have dual urban–rural systems.

The remainder of this paper is organized as follows: We introduce SoL approach and the variables used in our regression analyses in Section Method. Section Data presents descriptive statistics from our data. Section Results describes the empirical results of our analyses, including estimates of the extra costs for households with members who have a disability based on the SoL approach, and the rate of poverty in such households. In Section Discussion, we discuss the relationship between health insurance and the costs of disability as well as the policy-related implications of this research. Section Conclusion provides the main conclusions.

## Methods

The extra costs of disability mainly include direct and indirect costs (31). Direct costs refer to those incurred on assistive devices, medical rehabilitation, transportation, and related expenses. Indirect costs include reduced earnings by disabled people and the family members who take care of them. This article considers only the direct costs of disability. Several methods are available to estimate the direct costs (7, 32). First, expenditures that include extra goods and services for disabled persons are compared against those for people without disabilities. The differential is assumed to be the extra cost of disability (the goods and services-based approach or the comparative approach). But the goods and services-based approach is limited by the budgetary constraints of disabled people (33). Second, the extra costs include the market value of goods and services that are required by people with disabilities to perform particular activities (the subjective approach). The main problem with the subjective approach is that people with disabilities do not estimate the value of these activities if they are not aware that they can purchase some goods and services, and this leads to an underestimation of the extra costs for them (31). A third alternative is the standard of living approach. It identifies the difference between the incomes of people with disabilities and their non-disabled counterparts at the same living standard by constructing a regression of the SoL, income, and disability status. Researchers have used this approach increasingly commonly in recent years because it does not require measuring expenditure and is easy to use.

### Theoretical model

The standard of living approach assumes that people with disabilities have a lower standard of living than non-disabled persons with the same income. People with disabilities require additional goods and services to succeed in their environment (e.g., assistive devices, transportation costs, and daily care). The market value of these items is referred to as the extra costs of disability. For people with disabilities to have the same standard of living as those without disabilities, their income needs to be increased by an amount equal to the extra costs incurred. When measuring poverty, it is necessary to adjust income according to household characteristics, such as its size and composition, and the presence of persons with disabilities. Doing so equalizes the comparison of incomes across household types. Similar adjustments must be made when income is compared between households with and without members with disabilities to account for the extra costs of disability. The principle of the SoL approach is that the standard of living of a household is a function of income and demand. By controlling for the other variables, we can compare the standards of living of households with and without disabilities after accounting for the costs of disability.

The purpose of the SoL approach is to quantify how disability reduces the standard of living. This method was developed by Zaidi and Burchardt (9). The model is as follows:


(1) $$\begin{array}{l} S=\alpha Y+\beta D+\gamma X+k \end{array}$$

where *S* represents the standard of living for the household, *Y* is the household income, *D* is an indicator of disability, *X* are other household characteristics, and *k* indicates the minimum living standard. The extra costs of disability *E* can be derived from:


(2) $$\begin{array}{l} Y=\frac{1}{\alpha }S-\frac{\beta }{\alpha }D-\frac{\gamma }{\alpha }X-\frac{1}{\alpha }k \end{array}$$

(3) $$\begin{array}{l} E=\frac{dY}{dD}=-\frac{\beta }{\alpha } \end{array}$$

**Figure 1:**
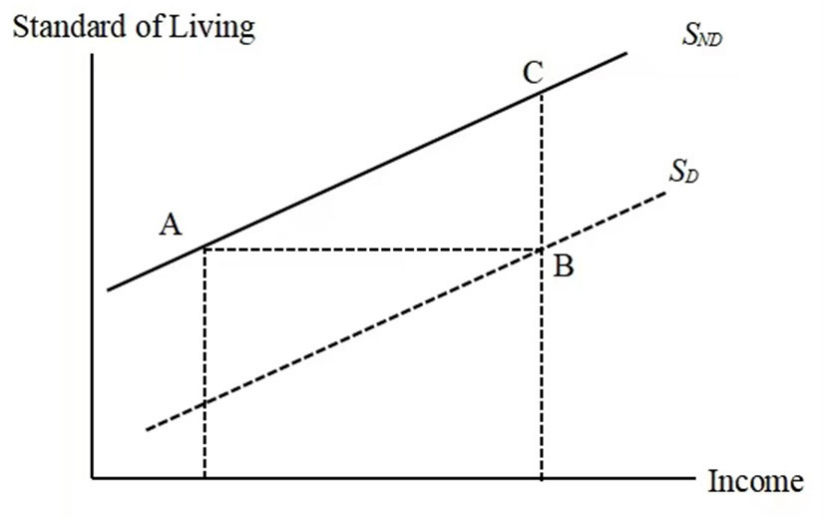
Income, standard of living and disability. (S_ND_ and S_D_ represent non-disabled and disabled people, respectively).

The measurement of the costs of disability based on the SoL approach is illustrated in Figure 1.

Under the above assumptions, Figure 1 shows that households with disabled members require income equal to the amount (*B*−*A*) to achieve the same standard of living as households without disabled members. If $\alpha =\frac{BC}{AB}$ is the slope of the standard of living of households without members with disabilities, and β is the distance between points *B* and *C*, then $\frac{\beta }{\alpha }\text{=}\frac{BC}{BC/AB}=AB$ represents the extra costs of disability. This means that the extra costs of disability can be determined algebraically and graphically by the ratio of the coefficients of disability and income from Equation (1). We can obtain the coefficients by estimating the regression described by Equation (1) above.

### Empirical model

We measure the financial costs of disability by using the following empirical model:


(4) $$\begin{array}{l} S=\delta +\alpha Hinc+\beta Dis+\gamma X+u_{0} \end{array}$$

*S* represents the SoL, *Hinc* represents the annual disposable household income in Chinese Yuan (CNY), *Dis* denotes disability status, *X* represents the control variables, and *u*_0_ is an error term. According to the SoL approach, the extra costs of disability can be determined by the ratio of the coefficients of disability and income, β*/*α.

Choosing appropriate proxy variables for the standard of living is the key to measuring the cost of disability by using the SoL approach. The choice of proxy variables needs to satisfy two requirements: (a) They must be independent of the status of disability, and be able to objectively reflect the SoL of all people in general. (b) They need to have sufficient income elasticity of demand. The latter point is important because if expenditures on necessities lack elasticity, this would mean that even high-income households would have a limited need for necessities and would not spend much income on them. Zaidi and Burchardt (9) selected household ownership of consumer durables, savings, and self-assessed financial situation as proxy variables. These variables are categorical. We follow their work, but with some differences: Our variables are continuous rather than categorical. The annual balance of financial assets is the SoL indicator, and is changed to savings in the sensitivity test.

## Data

### Data source

The data in this paper were taken from the Chinese Household Income Project Survey (CHIPS), which focuses on the income distribution and living conditions of China's urban and rural residents. The CHIPS applies a stratified multi-stage sampling approach to select the samples and data that are nationally representative. We used the data for 2008, 2013, and 2018 to explore the attributes of the extra costs of disability over time.

### SoL indicators

Two main approaches have been used in past work to choose the proxy variables of SoL indicators. One is the subjective wellbeing of the SoL, such as financial assessment (8, 15), and the other is objective measurement, such as the index or level of ownership of consumer durables (9, 12, 20, 24), and whether a household has any savings (9). We chose the annual balance of financial assets (BFA) of a household as the SoL indicator. The BFA consists of cash, savings, bonds, and stocks. It is a suitable choice as an SoL indicator because it can better represent financial stability than the subjective assessment of a household's financial situation. Subjective assessment is easily affected by the person's psychosocial status and thus becomes an unstable indicator of the living standard (15). Moreover, the BFA in these data was a continuous variable that contained more information about the financial situation of households than categorical variables.

### Independent variables

Although disability is defined generally by the International Classification of Functioning, Disability, and Health (ICF model), and is measured using activities of daily living (ADL) or instrumental activities of daily living (IADL), the official and social definitions of disability in China are still biomedical. To render our work here consistent with the situation in China, we used the medical model to define disability. The survey data provided only one way to define disability: specifically self-reported disability based on biomedical cognition. We measured the status of a disability through answers to the question “Do you have any disability (or chronic disease)?”^1^ The responses included (a) “No,” (b) “Yes, but no impact on normal work, study, or everyday activities,” and (c) “Yes, affecting normal work, study, or everyday activities.” Answers (b) and (c) were used to identify people with disabilities. Another crucial independent variable was household income, including the wages of all household members and the net business income. The relationship between household income and the SoL is not necessarily linear. Researchers usually assume that the costs of disability are proportional to the household income. This means the costs might rise with the household income, but with diminishing marginal effects of income on the standard of living (9). Therefore, *Hinc* in Equation (1) might be logarithmic income. Accordingly, we used the denary logarithm of annual disposable household income in the regressions. The characteristics of the head of the household are important control variables according to previous studies. Therefore, the age, education level, gender, marital status, and status as a minority of the heads of households were considered. The other covariates were composed of the number of children aged 0~17, household members beyond 60 years of age, participation in social insurance, and geographical location.

The descriptive statistics of the variables are shown in Table 1 by household type. We divided the data into those for non-disabled households (NDH) and disabled households (DH) by survey year, and performed *t*-tests on the mean values of the continuous variables and the chi-square test on the categorical variables. The basic descriptive statistics revealed substantial differences between NDH and DH. NDH had a higher income and balance of financial assets than DH did. The results of comparisons are in line with the findings of Wang et al. (23) where the mean annual income of households with older people with disabilities was found to be lower than that of their peers without disabilities in urban China. The mean educational level of the heads of households without disabilities was higher than that of heads of households with disabilities. The heads of DH were older than those of NDH, while DH had more members over 60 years of age. The overall coverage of basic pension insurance in China has been steadily advancing, and the participation of people with disabilities in social insurance has continued to increase in recent years. These factors have played an important role in reducing the economic burden on households. It should be noted that not everyone in China has social insurance. In general, people who work full time are required to participate in social insurance. However, those who do not have full-time jobs, such as rural residents and rural-to-urban migrants, may choose not to buy social insurance. To allow more people to obtain social security and build a social safety net for vulnerable groups, the Chinese government has been promoting its social insurance system in recent years, including subsidies for participants in rural insurance. Therefore, the portion of people participating in social insurance is increasing, but the extent of social security provided by rural social insurance remains limited.

**Table 1:** Descriptive statistics for variables by household type.

**Variables**	**2008**			**2013**			**2018**		
	**NDH**	**DH**	**Significance**	**NDH**	**DH**	**Significance**	**NDH**	**DH**	**Significance**
**Dependent variable**									
Balance of financial assets (mean)	¥58,254 (109,997)	¥37,166 (80,820)	***	¥60,366 (118,889)	¥34,548 (64,520)	***	¥106,874 (502,396)	¥81,786 (196,304)	***
**Independent variables**							
***Household head characteristics***							
Age	48 (10)	51 (10)	***	51 (12)	55 (12)	***	49 (12)	57 (12)	***
Education			***			***			***
Primary school and below	21%	34%		26%	43%		19%	31%	
Middle school	36%	42%		42%	39%		42%	40%	
High school	25%	18%		19%	12%		19%	19%	
College and above	18%	6%		13%	5%		20%	10%	
Male	81%	88%	***	84%	88%	***	80%	82%	***
Married	94%	95%	ns	90%	87%	***	91%	89%	***
Han	99%	99%	ns	94%	93%	**	94%	94%	*
***Households characteristics***							
Annual disposable household income	¥42,646 (49,336)	¥29,262 (26,433)	***	¥60,232 (56,311)	¥41,810 (36,887)	***	¥92,269 (79,742)	¥76,184 (79,724)	***
The number of children ages 0~17	0.67 (0 .73)	0.69 (0.75)	ns	0.62 (0.75)	0.57 (0.76)	**	0.75 (0.84)	0.58 (0.82)	***
The number of household members over 60 years old	0.30 (0.62)	0.57 (0.79)	***	0.52 (0.79)	0.83 (0.86)	***	0.43 (0.74)	0.99 (0.90)	***
Region			ns			***			***
Western	18%	20%		25%	28%		25%	31%	
Central	32%	32%		35%	39%		35%	40%	
Eastern	50%	48%		40%	33%		40%	29%	
***Social insurance participation***								
The number of household members participating in health insurance	3.36 (1.426)	3.93 (1.51)	ns	3.28 (1.40)	3.43 (1.52)	***	3.40 (1.40)	3.43 (1.61)	ns
The number of household members participating in pension insurance	0.85 (0.91)	0.53 (0.82)	ns	2.33 (1.29)	2.473 (1.36)	***	2.33 (1.24)	2.54 (1.29)	***
**Observations**	7,403	865	8,268	14,388	1,527	15,915	10,402	6,838	17,240

Bivariate significance (NDH vs. DH) determined by chi-square test on the categorical variables and t-test for continuous variables; ns = not significant. Standard errors in parentheses. *p < 0.1, **p < 0.05, ***p < 0.01.

## Results

### Extra costs of households containing members with disabilities

Table 2 presents the results of regression when the balance of household financial assets was considered a proxy variable of the standard of living. Based on these estimations, we used the SoL approach to calculate the extra costs incurred by households with PWD. The controlled variables include the characteristics of the head of household and the household itself, as previously mentioned. The results indicate that all coefficients based on the log household incomes were statistically significant. Meanwhile, all coefficients of households with disabled members were statistically lower than zero. This means that disability does reduce the households' standard of living, *ceteris paribus*.

**Table 2:** The extra costs of living with disability by urban-rural areas: The balance of household financial assets is a SoL index.

**Year**	**Rural**			**Urban**			**Rural**	**Urban**
**Location**	**2008**	**2013**	**2018**	**2008**	**2013**	**2018**	**2008–2018**	
Annual disposable household income, log	0.418***	0.812***	0.604***	0.525***	1.088***	0.823***	0.643***	0.800***
	(0.026)	(0.024)	(0.027)	(0.041)	(0.030)	(0.030)	(0.015)	(0.018)
Households with disabled members	−0.147**	−0.332***	−0.267***	−0.204**	−0.179***	−0.203***	−0.191***	−0.225***
	(0.061)	(0.047)	(0.038)	(0.086)	(0.063)	(0.036)	(0.026)	(0.028)
*Extra costs*	35.1%	40.8%	44.1%	38.9%	16.4%	24.6%	29.7%	28.1%
Observations	4,484	8,633	8,411	3,784	7,282	8,829	21,528	19,895
Adjusted R^2^	0.201	0.253	0.192	0.269	0.306	0.207	0.222	0.239

The control variables are the number of children ages 0~17, the number of household members over 60 years old, region, the number of household members participating in medical insurance and pension insurance, and the characteristics of head of household including age, education level, gender, marital status, minority. For saving space, they are not listed in the table. Standard errors in parentheses. *p < 0.1, **p < 0.05, ***p < 0.01.

For rural households with PWD, the extra costs of disability have increased from 2008 to 2018, while for urban households with PWD, they fell first in 2013 and then rose in 2018. Note that the gap in the costs of disability between urban and rural areas in the opposite manner in the same period. At the beginning, the costs of disability were higher for urban households compared with rural households. Then, the costs for urban households became less than those for rural households in 2013, and this trend persisted up to 2018. Although the costs of disability increased for both rural and urban households in 2018 compared with those in 2013, the gap between them had shrunk. This might reflect the tendency of convergence of urban–rural disability costs.

Another notable result is that the average costs of disability were higher for rural households than for urban households. On average, the share of extra costs of disability in rural areas (29.7%) was slightly larger than that in urban areas (28.1%). This result is different from the estimations of Loyalka et al. (24), who claimed that the costs of disability were higher for urban households than for rural households in China. They did not present the trend of changes in the costs of disability over time because their data was limited to 2006. Our findings demonstrate that the costs of disability were higher for rural households than for urban households in 2013 and 2018. It is also worth noting that in our regressions of 2008, close in time to the year considered by Loyalka et al. (24) the estimations agreed with their results. However, the trend changed in 2013, and the results show that the costs of disability were considerably higher for rural households than for urban households (40.8 vs. 16.4%). Significantly, the urban–rural gap in the extra costs of disability in 2013 was different from that 5 years ago, and this situation persisted up to 2018. Therefore, the results show, at the very least, that the extra costs of disability in urban areas were not always higher than those in rural areas.

### Poverty rate of households with disabled members

**Figure 2:**
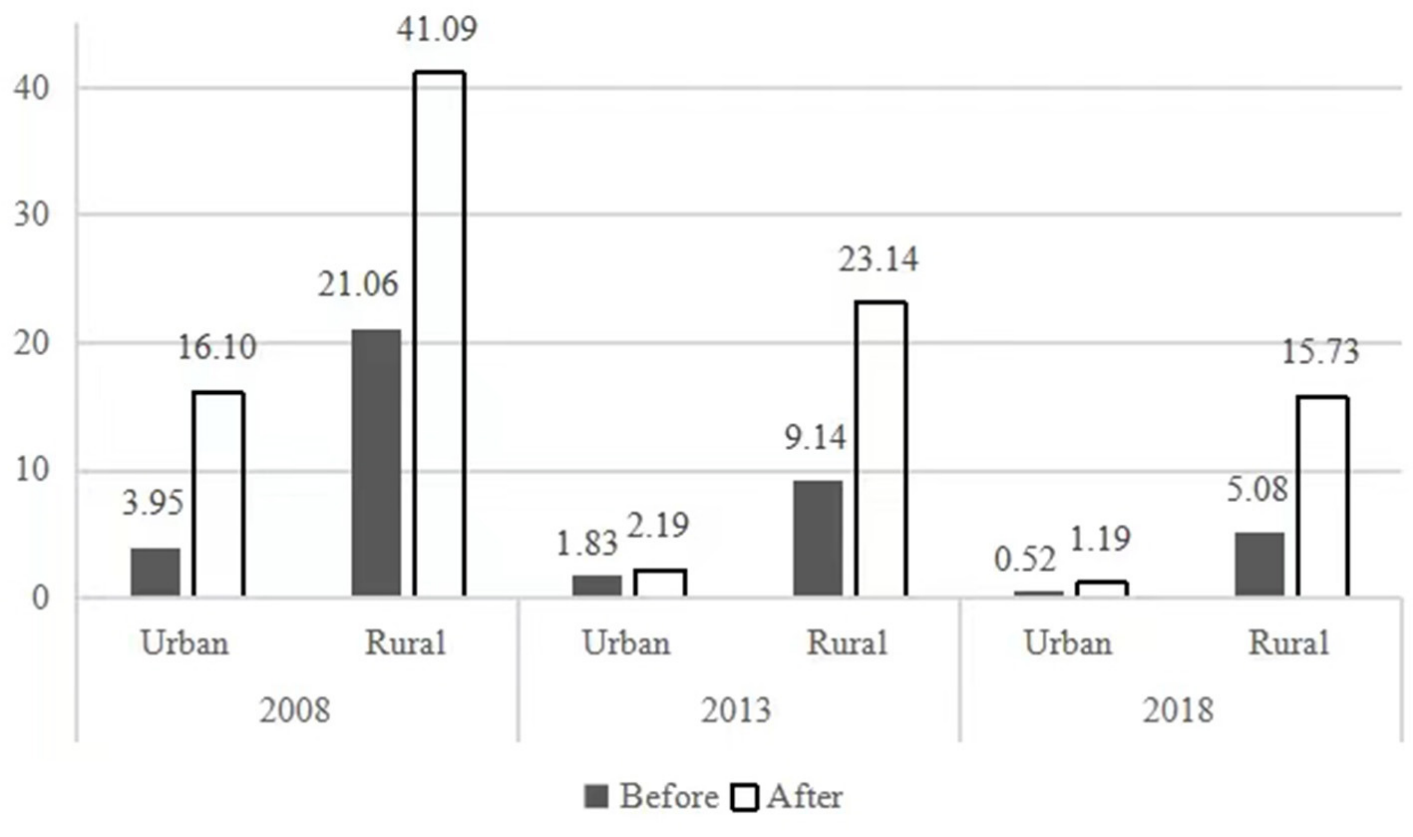
The rates of poverty of households with disabled people before and after income equivalence adjustment.

The rate of poverty of households with disabled people rises once the extra costs of disability are taken into account, which means income equivalence adjustment. Figure 2 shows the rates of poverty of households containing people with disabilities before and after equivalence adjustment in both urban and rural areas from 2008 to 2018. We used the currently used poverty alleviation standard of CNY2,300 per person per year issued in 2011, and this was equivalent to CNY2,242 in 2008, CNY2,736 in 2013, and CNY2,995 in 2018 based on the CPI. The Foster–Greer–Thorbecke (FGT) Poverty Index was applied to measure the poverty rate in this paper. The rates of poverty of all the households rose after the income equivalence adjustment, especially that of rural households with disabled people. From the perspective of time, the poverty rates of urban and rural households with disabled people have fallen steadily. As a whole, rural households with disabled people experienced much greater poverty rate than urban households in the three survey years considered, regardless of whether the extra costs of disability were considered.

### Sensitivity tests

We used the logarithm of household savings as the proxy variable of the SoL in our sensitivity tests and controlled the same independent variables as were previously regressed. The findings were similar: The trend of change in of the urban–rural gap in the extra costs of disability were similar (see Table 3). In 2008, the extra costs of disability were lower for rural households than for urban households, whereas the relationship was the reverse of this in 2013 and 2018. These results exhibited stable trend to this effect. The estimated results were also sensitive to the various proxy variables of the SoL. On average, the share of extra costs of disability for rural households (35.6%) was slightly higher than for urban households (34.6%), which is similar to the result of the balance of household financial assets as a SoL index.

**Table 3:** The extra costs of living with disability by urban-rural areas: Household savings are a SoL index.

**Year**	**Rural**			**Urban**			**Rural**	**Urban**
**Location**	**2008**	**2013**	**2018**	**2008**	**2013**	**2018**	**2008–2018**	
Annual disposable household income, log	0.332***	0.791***	0.590***	0.463***	0.988***	0.708***	0.614***	0.704***
	(0.027)	(0.024)	(0.027)	(0.038)	(0.030)	(0.029)	(0.015)	(0.018)
Households with disabled members	−0.111*	−0.329***	−0.250***	−0.226***	−0.200***	−0.210***	−0.197***	−0.244***
	(0.062)	(0.047)	(0.038)	(0.083)	(0.064)	(0.036)	(0.026)	(0.028)
*Extra costs*	33.4%	41.6%	42.3%	48.8%	20.3%	29.7%	35.6%	34.6%
Observations	3,777	8,604	8,389	3,633	7,253	8,792	20,770	19,678
Adjusted R^2^	0.179	0.241	0.187	0.227	0.262	0.158	0.206	0.191

The control variables in this table are the same as those in Table 2. We do not list them for saving space. Standard errors in parentheses. *p < 0.1, **p < 0.05, ***p < 0.01.

## Discussion

### Expenditure on healthcare, and coverage of health insurance and rehabilitation services for people with disabilities

The apparent reversal of the urban–rural gap in the extra costs of disability from 2008 to 2018 leads us to explore reasons for it. The expenditure on healthcare is the most significant additional costs for the households with PWD. According to 2008–2013 reports on the situation of persons with disabilities in China, out-of-pocket expense on healthcare by households with PWD was more than double that for households without PWD, and was the second-largest expenditure for PWD.

We used data from the 2008–2013 longitudinal survey of disabled persons and the 2018 National Survey on the Income of Families with Disabled Persons to analyze the available services for PWD and their incomes both in urban and rural areas. These surveys were conducted by China's Disabled Persons' Federation, and their aim was to collect information on disabled persons' living, and their development and environment. We selected several variables at the household level related to the standard of living and the extra costs of disability, including disposable income, expenditure on healthcare, and the percentage of PWD participating in health insurance and at least one of the rehabilitation services on offer. As shown in Table 4, changes in the expenditure on healthcare as a percentage of disposable income were in accord with the trend of extra costs of disabilities over time. The disposable income as well as the expenditure on health increased from 2008 to 2018, where this growth was faster for rural households than urban households. Accordingly, the expenditure on healthcare as a percentage of disposable income for rural households rose over the period, while it fell first and then rose for urban households with PWD. This tendency was in line with the variation in the extra costs of disability mentioned above.

**Table 4:** Income, expenditure, and rehabilitation services for PWD.

	**Disposable income (CNY)**		**Expenditure on health care (CNY)**		**Expenditure on health care as a percentage of disposable income**		**A percentage of PWD on participation in health insurance**		**A percentage of PWD on participation in at least one of rehabilitation services**	
**Year**	**Urban**	**Rural**	**Urban**	**Rural**	**Urban**	**Rural**	**Urban**	**Rural**	**Urban**	**Rural**
2008	8,487.2	3,803.6	1,150	449	13.5%	11.8%	58.6%	93.5%	36.6%	19.2%
2013	15,851.4	7,829.9	1,789	1,033	11.3%	13.2%	93.7%	97.1%	64.8%	56.1%
2018	22,413.3	13,364.9	3,662	2,276	16.3%	17.0%	/	/	/	/

There is no information about participation in at least one of rehabilitation services for PWD in 2018 survey.

China's urban and rural areas have different health insurance schemes, with different rates of participation. The three types of health insurance schemes currently implemented in China are all government led. First, the Urban Employee Basic Health Insurance (UEBHI) scheme covers employees and retirees from the formal urban sector (compulsory). It began in the late 1990's. Second, the Urban Resident Basic Health Insurance (URBHI) scheme covers unemployed residents and children in urban areas (voluntary), and was launched in 2007. Third, the New Cooperative Medical Scheme (NCMS) covering rural residents (voluntary) was piloted in 2003 and fully implemented in 2010 (34). Until 2018, these three schemes covered a majority of the Chinese population. In urban China, the ratio of disabled persons over 16 years of age who participated in the Urban Employee/Resident Basic Health Insurance increased rapidly, from 58.6% in 2008 to 93.7% in 2013. This is why the additional costs of urban PWD decreased quickly. From 2013 to 2018, the costs for urban disabled people rebounded. There may be two likely explanations for this. One is that the cost of the same healthcare services rose, and the other is that people with disabilities began receiving more kinds of services than before.

There are two possible explanations for why extra costs have been rising for rural households with PWD from 2008 to 2018. On the one hand, access to healthcare and rehabilitation services grew such that rural PWD could avail themselves of more services, because of which the costs of these services increased. On the other hand, the reimbursement for health insurance for rural PWD did not rise markedly in this period, because the ratio of rural disabled people participating in NCMS did not expand by much, and their ratio of reimbursement did not raise significantly in this period.

By comparison, the extra costs of disability for rural PWD were lower than those for urban PWD in 2008, but were higher in 2013 and 2018. Rural PWD had less access to rehabilitation services in 2008 compared with urban PWD (the last two columns of Table 4). Moreover, with social and economic development, rural PWD were likely to access some services that they could not access before such that the difference in their access to rehabilitation services with urban PWD decreased. As a result, rural PWD spent more on healthcare and rehabilitation services in 2013 and 2018. In addition, the rate of reimbursement of the NCMS was much lower than that of the UEBHI and URBHI. This led to higher out-of-pocket expenditure by rural PWD than urban PWD.

### Policy implications

Our study has two important policy implications. On the one hand, the extra costs of disability need to be taken into account when measuring poverty. The rate of poverty of households with PWD increased when disability costs were considered, where this rise was higher for rural households than for urban households. This implies that the extra costs of disability affected poorer households more than richer ones (9). High costs of disability might be a critical reason for why households with PWD have fallen into poverty. Policymakers need to pay more attention to poor populations with disabilities, and consider the costs of disability when assessing household poverty. The alternative is extending health insurance coverage or providing more free services for PWD to reduce disability costs. As has been previously analyzed, the additional costs for urban disabled people decreased (2013 vs. 2008) when their health insurance coverage was enhanced. Although the accessibility of rural households with PWD to rehabilitation services has risen considerably in recent years, the quality and coverage of services remains limited in rural China. For example, doctors in rural areas may not be highly skilled at diagnosis and treatment, and may not have suitable medical equipment available. In terms of coverage, some rehabilitation services remain difficult to reach in remote mountainous areas.

On the other hand, countries with dual urban–rural systems should provide equitable public health services. Although health insurance coverage is now relatively widespread in China, it remains low at the per capita level. More importantly, the reimbursement rate for health insurance in rural areas is much lower than that in urban China. Unequal health insurance schemes exacerbate the gap in extra costs for PWD between urban and rural areas. Once the costs of disability are considered, the gap in the rate of poverty between urban and rural households with PWD widens, even if rural households with PWD have seen incomes and opportunities of access to rehabilitation services rise at a faster rate than urban households with PWD have. Therefore, an equalization of publicly-funded health services between urban and rural areas is the foundation on which people with disabilities in rural areas can build to truly benefit from economic development and improve their living standards.

## Conclusion

In this study, we found that the extra costs of disability in urban and rural China have changed differently over time. The work here represents a significant improvement over previous studies in the area through the inclusion of data from three time points from surveys over the last 10 years. Unlike past studies, we found that the costs of disability in rural areas were not always consistently lower than in urban areas, and were closely related to health insurance. By taking the costs of disability into account, we found that the adjusted poverty rates of households with PWD were higher than before, where this increase was greater in rural areas than in urban areas. Further, the adjusted poverty rate of the entire population rose due to the costs of disability.

The main contributions of our work are twofold. First, this research provides information on changes in the urban–rural gap in the costs of disability over time. The results did not exhibit a consistent tendency over time with regard to the extra costs of disability between urban and rural areas in China. This variance reflects the inequity in medical insurance and healthcare services between urban and rural areas. It suggests that policy makers should establish more equitable and effective public health systems for both urban and rural residents. Second, the results challenge the stereotype that the extra costs of disability in rural areas are lower than those in urban areas, or that these costs in low- or middle-income countries are lower than those in high-income countries, as has been claimed in previous studies. Mitra et al. (31) reviewed research on the extra costs of disabilities from different countries, and claimed that the findings provided initial evidence that the costs of disability were higher as a proportion of household income in high-income countries relative to low- and middle-income countries. This research presented different findings from data on three measurement points over 10 years. The urban–rural gap in the costs of disability has accentuated the inequity between urban and rural people with disabilities. Therefore, it is necessary to raise the rate of reimbursement for health insurance for rural disabled people while expanding the rehabilitation services for them.

This study has two main limitations. One is that the definition of disability was based on self-report, which lowers the rate of disability (35). This likely leads to an underestimation of the rate of poverty in the entire population. This situation reminds us to give due consideration to the extra costs of disability and their effects. The second shortcoming of this work is the use of cross-sectional data, which cannot control for unobserved heterogeneity and the dynamics of disability. The panel model has advantages over the cross-section model, such as control for intra-individual effects, and lagged levels of disability and income (12). Considering the benefit of the capability for comparisons with international work, future research would be best advised to use the Washington Group Short Set of Questions on Disability to measure status of disability in China (36). Panel data are also a better choice for studying the extra costs of disability and their effects on poverty in China in the future.

## Data availability statement

The datasets presented in this article are not readily available because the data that support the findings of this study are available from the corresponding author upon reasonable request. Requests to access the datasets should be directed to QW, wq68986@163.com.

## Author contributions

JL designed the study, collected the data, conducted the data analysis, and wrote the original draft. QW conducted the data analysis, helped write, and review the manuscript. J-LH explored the proposed method and conducted data analysis. Y-MW contributed to the writing, reviewing, and editing of the manuscript. All authors contributed to the article and approved the submitted version.

## Funding

The work was supported by Grants (17YJAZH051) from Humanities and Social Sciences Project of the Ministry of Education's Planning Fund for the People's Republic of China and Grants (22&ZZ003) from China Disabled Persons' Federation.

## Conflict of interest

The authors declare that the research was conducted in the absence of any commercial or financial relationships that could be construed as a potential conflict of interest.

## Publisher's note

All claims expressed in this article are solely those of the authors and do not necessarily represent those of their affiliated organizations, or those of the publisher, the editors and the reviewers. Any product that may be evaluated in this article, or claim that may be made by its manufacturer, is not guaranteed or endorsed by the publisher.

## Footnotes

^1^The question in the 2008 and 2013 questionnaires was “Do you have any disability?” and that in the 2018 questionnaire was “Do you have any disability or chronic disease?”
